# Manifestations and Outcomes of Intracerebral Hemorrhage During the COVID-19 Pandemic in China: Multicenter, Longitudinal Cohort Study

**DOI:** 10.2196/34386

**Published:** 2023-12-13

**Authors:** Yan Wan, Quan Wei He, Shaoli Chen, Man Li, Yuanpeng Xia, Lei Zhang, Zhou Sun, Xiaolu Chen, David Wang, Jiang Chang, Bo Hu

**Affiliations:** 1 Department of Neurology, Union Hospital Tongji Medical College Huazhong University of Science and Technology Wuhan China; 2 Barrow Neurological Institute St. Joseph's Hospital and Medical Center Phoenix, AZ United States; 3 School of Public Health, Tongji Medical College Huazhong University of Science and Technology Wuhan China

**Keywords:** COVID-19, intracerebral hemorrhage, manifestation, outcome, cohort study

## Abstract

**Background:**

The COVID-19 pandemic has inevitably affected the distribution of medical resources, and epidemic lockdowns have had a significant impact on the nursing and treatment of patients with other acute diseases, including intracerebral hemorrhage (ICH).

**Objective:**

This study aimed to investigate how the COVID-19 pandemic affected the manifestations and outcomes of patients with ICH.

**Methods:**

Patients with acute ICH before (December 1, 2018-November 30, 2019) and during (December 1, 2019-November 30, 2020) the COVID-19 pandemic at 31 centers in China from the Chinese Cerebral Hemorrhage: Mechanism and Intervention (CHEERY) study were entered into the analysis. Demographic information and clinical manifestations and outcomes were collected and compared between the 2 groups.

**Results:**

From December 1, 2018, to November 30, 2020, a total of 3460 patients with ICH from the CHEERY study were enrolled and eventually analyzed. The results showed that during the COVID-19 pandemic, patients with ICH were more likely to be older (*P*<.001) with a history of ischemic stroke (*P*=.04), shorter time from onset to admission (*P*<.001), higher blood pressure (*P*<.001), higher fasting blood glucose (*P*=.003), larger hematoma volume (*P*<.001), and more common deep ICH (*P*=.01) and intraventricular hemorrhage (*P*=.02). These patients required more intensive care unit treatment (*P*<.001) and preferred to go to the hospital directly rather than call an ambulance (*P*<.001). In addition, the COVID-19 pandemic was associated with an increased risk of pulmonary infection during hospitalization (adjusted risk ratio [RR_adjusted_] 1.267, 95% CI 1.065-1.509), lower probability of a 3-month good outcome (RR_adjusted_ 0.975, 95% CI 0.956-0.995), and a higher probability of in-hospital (RR_adjusted_ 3.103, 95% CI 2.156-4.465), 1-month (RR_adjusted_ 1.064, 95% CI 1.042-1.087), and 3-month (RR_adjusted_ 1.069, 95% CI 1.045-1.093) mortality.

**Conclusions:**

Our study indicated that the cloud of COVID-19 has adversely impacted the presentation and outcomes of ICH. Medical workers may pay more attention to patients with ICH, while the public should pay more attention to hypertension control and ICH prevention.

**Trial Registration:**

Chinese Clinical Trial Registry ChiCTR1900020872; https://www.chictr.org.cn/showprojEN.html?proj=33817

## Introduction

COVID-19, the pneumonia caused by SARS-CoV-2 infection, emerged in Wuhan City, Hubei Province, China in December 2019 [1]. The World Health Organization (WHO) declared the COVID-19 outbreak a public health emergency of international concern on January 30, 2020, and declared a pandemic on March 11, 2020 [2]. In China, from January 3, 2020, to March 16, 2023, there have been 99,185,059 confirmed cases of COVID-19 with 120,576 deaths reported to the WHO [3]. Wuhan was the core area of the COVID-19 storm in China, and this city was locked down from January 23, 2020, to April 8, 2020 [4]. Various public health interventions were implemented by the Chinese government to suppress the big pandemic, including but not limited to intensive intracity and intercity traffic restrictions, social distancing measures, home isolation and centralized quarantine, and improvement of medical resources [5,6].

In addition, as a global health crisis, the COVID-19 pandemic has been the most serious challenge to delivering timely care to patients with other conditions [7]. Its outbreak proved to have a major impact on patients with cerebrovascular diseases [8,9], for example, significantly decreasing the rate of stroke admissions [10,11], intravenous thrombolysis and mechanical thrombectomy [12,13], and prolonging the time from symptom onset to treatment of ischemic stroke [14]. Intracerebral hemorrhage (ICH), one of the most devastating diseases worldwide with a mortality rate of up to twice that of ischemic stroke [15], requires emergency medical care in specialized neurological intensive care units [16]. Under the condition of tight medical resources, it was difficult to balance stroke treatment and COVID-19 prevention during the COVID-19 pandemic [8-10,12], and it is not clear whether the COVID-19 pandemic affected the manifestations and outcomes of patients with ICH.

Moreover, hypertension management during the COVID-19 pandemic may be a challenge, which is an important therapy for ICH [17,18]. There is an unproven notion that angiotensin-converting enzyme inhibitors (ACEIs) and angiotensin receptor blockers (ARBs) might theoretically increase the risk of COVID-19 infection because increased angiotensin-converting enzyme-2 activity has been shown to lead to practical medicine restriction. Recently, there have been reports that discontinuation of ACEI/ARB in patients with COVID-19 is not related to the severity of COVID-19 [19,20]. However, discontinuation of ACEI/ARB was once not an uncommon phenomenon. Besides, during the COVID-19 pandemic, the public may have also suffered from social pressure, anxiety, the depressed economy, a lack of public health resources, and so on [21,22]. All of these factors may disturb the control of risk factors of ICH, resulting in increased occurrence and poor outcomes.

The Chinese Cerebral Hemorrhage: Mechanism and Intervention (CHEERY) study is a prospective multicenter cohort study that began on December 1, 2018, and recruits patients with spontaneous ICH within 7 days of onset in China [18]. This cohort covered periods before and after the COVID-19 outbreak. In this study, in order to investigate how the COVID-19 pandemic affected the manifestations and outcomes of patients with ICH, we retrospectively analyzed the data of patients with ICH in 1-year periods before (December 1, 2018-November 30, 2019) and during (December 1, 2019-November 30, 2020) the COVID-19 pandemic in the CHEERY study.

## Methods

### Study Design

The CHEERY study is a multicenter Chinese program that includes consecutive patients with ICH admitted to 31 stroke centers beginning in December 2018. It recruits patients with spontaneous ICH within 7 days of onset and aims to identify any major risk factors affecting the prognosis of ICH [18]. To explore the effects of the COVID-19 pandemic on the manifestations and outcomes of patients with ICH, we selected and analyzed data from December 1, 2018, to November 30, 2020, which happens to span 1 year before (December 1, 2018-November 30, 2019) and during (December 1, 2019-November 30, 2020) the epidemic. We followed the STROBE (Strengthening the Reporting of Observational Studies in Epidemiology) reporting guidelines.

### Participants

All patients with ICH enrolled in the study must meet the following criteria: (1) older than 18 years; (2) have acute ICH with ≤7 days duration from onset to admission; and (3) have received either computerized tomography or magnetic resonance imaging of the brain to confirm the diagnosis of ICH. Due to the centralized isolation of patients with COVID-19, this study did not include patients with ICH complicated with COVID-19 infection.

### Data Collection and Analysis

Demographic information was collected, including age, gender, residence, smoking and alcohol history, and medical history from the medical record. Gender was recorded from the medical record, which was copied from the patient’s resident ID card. The mode of transportation to the hospital and duration from onset to admission were collected shortly after admission from patients or escorts. Systolic blood pressure, diastolic blood pressure, hematoma location, hematoma volume, and laboratory data were copied from the medical record. The baseline National Institute of Health Stroke Scale and Glasgow coma scale were assessed by 2 trained neurologists. After admission, the first head computerized tomography scan was acquired, and 2 imageologists calculated the volume of bleeding with the ABC/2 formula and recorded the location of bleeding and intraventricular hemorrhage (IVH). The SMASH-U (structural vascular lesions, medication, amyloid angiopathy, systemic disease, hypertension, and undetermined) flowchart was adopted to classify the most likely cause for each ICH event [23]. Structural vascular lesions were defined as vascular structural abnormalities at the bleeding sites verified by imaging or pathological findings. Medication was defined as warfarin use with an international normalized ratio ≥2, novel oral anticoagulant use within 3 days, full-dose heparin, or thrombolytic agent use. Amyloid angiopathy was defined as lobar, cortical, or subcortical hemorrhage among patients aged ≥55 years, according to the Boston criteria. Systemic disease was conventionally defined by the presence of thrombocytopenia or liver cirrhosis or alternatively by the presence of non–drug-induced coagulopathy or renal failure. Hypertension was defined as deep or infratentorial hemorrhage with pre-existing hypertension history. All other causes and unknown causes were considered undetermined. Pulmonary infection was diagnosed by 2 well-trained and experienced neurologists according to the modified Centers for Disease Control and Prevention criteria in combination with the patient’s clinical symptoms, laboratory results, and radiological examinations within 1 week after ICH. A modified ranking scale score was assessed centrally by face-to-face, telephone, or WeChat interview.

### Definitions

ICH was defined as hemorrhage into the cerebral parenchyma that may also extend into ventricles, and rarely, subarachnoid spaces. A modified ranking scale score of 0-3 points was defined as a good outcome.

### Statistical Analysis

Data were presented as the mean (SD), number (%), and median (IQR). The Kolmogorov-Smirnov test for normality was used to assess the data distribution of continuous variables. Continuous variables were tested with a *t* test or Mann-Whitney U test. Categorical variables were tested with the χ^2^ test or Fisher exact test. The associations of the outcomes of interest between the 2 groups were analyzed using the modified Poisson regression model, and risk ratios (RRs) were reported along with 95% CIs. Age and gender were used as covariables. SPSS statistical software (version 26.0, SPSS Corp) was used to analyze the data, and the statistical significance was set at *P*<.05.

### Ethical Considerations

The study protocol and data collection were conducted strictly following the Declaration of Helsinki and approved by the Research Ethics Committee of Tongji Medical College, Huazhong University of Science and Technology, Wuhan, China (approval no. 2018-S485) and registered in the Chinese Clinical Trial Registry (ChiCTR1900020872) [24]. Protocol training was conducted for all researchers prior to the implementation of the project. All participants signed a written informed consent form prior to enrollment. The informed consent should be signed by the participant, but for those participants who could not sign the informed consent form by themselves for any reason, their parents, legal guardians, or protectors signed the informed consent form.

## Results

**Figure 1:**
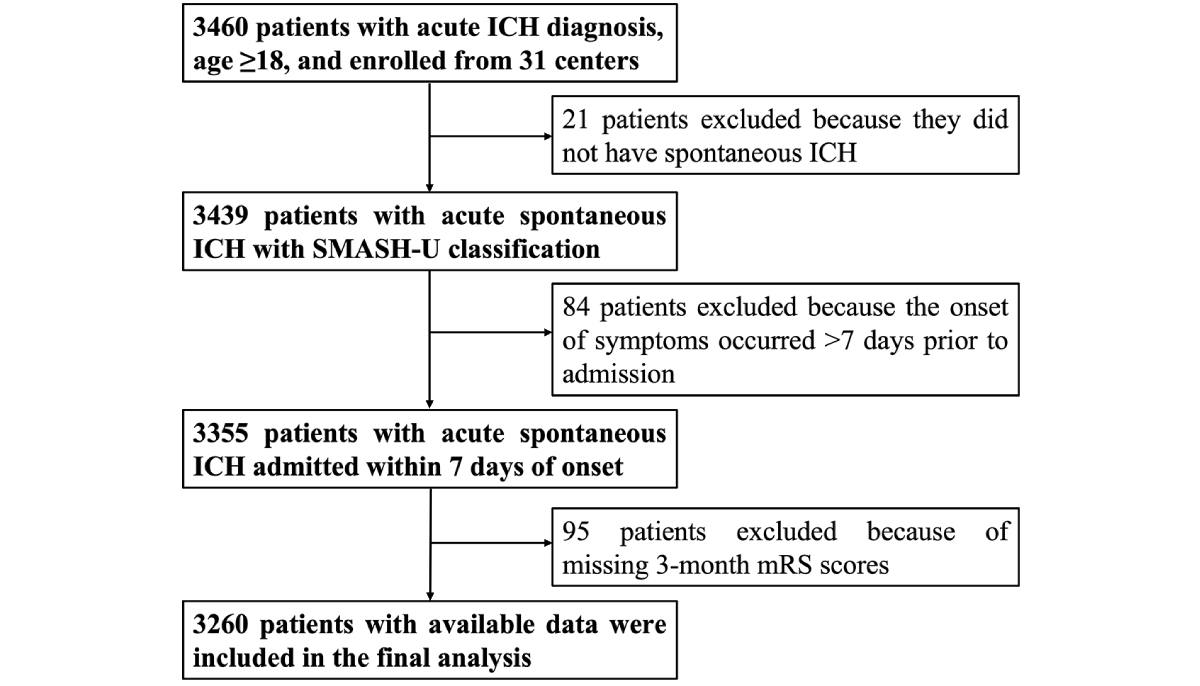
Flowchart of patient enrollment in the Chinese Cerebral Hemorrhage: Mechanism and Intervention study. ICH: intracerebral hemorrhage; mRS: modified ranking scale; SMASH-U: structural vascular lesions, medication, amyloid angiopathy, systemic disease, hypertension, and undetermined.

From December 1, 2018, to November 30, 2020, there were 3460 patients with acute ICH enrolled in the CHEERY study, and 3260 patients were included in the final analysis (Figure 1). The demographic data are listed in Table 1.

**Table 1:** Baseline and admission characteristics of patients admitted to hospitals in the year during COVID-19 pandemic (December 1, 2019-November 30, 2020) and the year before the COVID-19 outbreak (December 1, 2018-Novemver 30, 2019).

Characteristic		Time period		*P* value
		During the COVID-19 pandemic (n=1510)	Before the COVID-19 pandemic (n=1750)	
Age (years), median (IQR)		63 (54-71)	61 (52-70)	<.001
**Age (years), n (%)**				.001
	<60	625 (41.4)	828 (47.3)	
	≥60	885 (58.6)	922 (52.7)	
**Gender, n (%)**				.009
	Men	1045 (69.2)	1136 (64.9)	
	Women	465 (30.8)	614 (35.1)	
**Residence, n (%)**				
	Rural	850 (56.3)	930 (53.1)	.18
	Urban	639 (42.3)	797 (45.5)	
	Unknown	21 (1.4)	23 (1.3)	
**Smoking history, n (%)**				.001
	Yes	424 (28.1)	584 (33.4)	
	No	1081 (71.6)	1159 (66.2)	
	Unknown	5 (0)	7 (0)	
**Alcohol history, n (%)**				.02
	Yes	346 (22.9)	464 (26.5)	
	No	1159 (76.8)	1279 (73.1)	
	Unknown	5 (0)	7 (0)	
**Comorbidities, n (%)**				
	Ischemic heart disease	62 (4.1)	53 (3)	.25
	Ischemic stroke	156 (10.4)	150 (8.6)	.04
	Hypertension	970 (64.7)	1141 (66.1)	.40
	Diabetes	137 (9.1)	143 (8.2)	.06
Antihypertension^a^		281/970 (29)	299/1141 (26.2)	.16
Pre-ACEI/ARB^b^ usage		49/281 (17.4)	45/299 (15.1)	.44
**Transportation to hospital, n (%)**				<.001
	Ambulance	660 (43.7)	1012 (57.8)	
	Self-admission	838 (55.5)	686 (39.2)	
	Unknown	12 (0.8)	52 (3)	
Duration from onset to admission (hours), median (IQR)		4 (2-12)	6 (2-24)	<.001
SBP^c^ on admission (mm Hg), median (IQR)		170 (150-190)	162 (145-180)	<.001
DBP^d^ on admission (mm Hg), median (IQR)		97 (87-108)	94 (84-106)	<.001
Fasting blood glucose (mmol/L), median (IQR)		6.3 (5.3-7.9)	6.2 (5.3-7.4)	.003
NIHSS^e^ on admission, median (IQR)		9 (4-16)	8 (3-15)	.05
GCS^f^ on admission, median (IQR)		14 (10-15)	14 (11-15)	.009
**Location of hematoma, n (%)**				.01
	Nondeep	292 (19.3)	403 (23)	
	Deep	1218 (80.7)	1347 (77)	
Hematoma volume (ml), median (IQR)		12.0 (5.0-28.0)	10.7 (4.9-22.0)	<.001
IVH^g^, n (%)		249 (16.5)	238 (13.6)	.02
ICU^h^ treatment, n (%)		960 (63.7)	938 (55.2)	<.001
Surgical intervention, n (%)		271 (17.9)	358 (20.5)	.07
**SMASH-U^i^ classification, n (%)**				
	Structural lesion	26 (1.9)	31 (1.8)	.83
	Medication	7 (0.5)	4 (0.2)	.19
	Amyloid angiopathy	196 (14.7)	235 (13.9)	.58
	Systemic diseases	16 (1.2)	18 (1.1)	.74
	Hypertension	725 (54.2)	919 (54.5)	.86
	Undetermined	361 (27)	478 (28.4)	.41
Hospitalization length (days), median (IQR)		15 (8-21)	15 (10-21)	.02

^a^Percentage of pre-ICH antihypertension treatments in patients with hypertension.

^b^ACEI/ARB: angiotensin-converting enzyme inhibitor/angiotensin receptor blocker.

^c^SBP: systolic blood pressure.

^d^DBP: diastolic blood pressure.

^e^NIHSS: National Institute of Health Stroke Scale.

^f^GCS: Glasgow coma scale.

^g^IVH: intraventricular hemorrhage.

^h^ICU: intensive care unit.

^i^SMASH-U: structural vascular lesions, medication, amyloid angiopathy, systemic disease, hypertension, and undetermined.

The patients’ median age was 63 (IQR 54-71) years during the COVID-19 pandemic and 61 (IQR 52-70) years before the COVID-19 breakout (*P*<.001). The COVID-19 pandemic was associated with a higher percentage of male patients (*P*=.009) and patients with a history of ischemic stroke (*P*=.04). Fewer patients had a history of smoking (*P*=.001) and drinking (*P*=.02) during the pandemic (Table 1).

During the COVID-19 pandemic, patients were more likely to go to the hospital directly by themselves (during: n=838, 55.5%; before: n=686, 39.2%; *P*<.001) and took less time from onset to admission (during: median 4, IQR 2-12 hours; before: median 6, IQR 2-24 hours; *P*<.001) compared with before the pandemic. The COVID-19 pandemic was also associated with an increase in systolic blood pressure (during: median 170, IQR 150-190 mm HG; before: median 162, IQR 145-180 mm Hg; *P*<.001), diastolic blood pressure (during: median 97, IQR 87-108 mm HG; before: median 94, IQR 84-106 mm Hg; *P*<.001), fasting blood glucose (during: median 6.3, IQR 5.3-7.9 mmol/L; before: median 6.2, IQR 5.3-7.4 mmol/L; *P*=.003), deep ICH (during: n=1218, 80.7%; before: n=1347, 77%; *P*=.01), hematoma volume (during: median 12.0, IQR 5.0-28.0 mL; before: median 10.7, IQR 4.9-22.0 mL; *P*<.001), intraventricular hemorrhage (during: n=249, 16.5%; before: n=238, 13.6%; *P*=.02), and ICU treatment (during: n=960, 63.7%; before: n=938, 55.2%; *P*<.001) (Table 1).

We then comparatively evaluated the outcomes of ICH. Table 2 shows that the COVID-19 pandemic was associated with an increased risk of pulmonary infection during hospitalization (RR_unadjusted_ 1.299, 95% CI 1.091-1.546; RR_adjusted_ 1.267, 95% CI 1.065-1.509), lower probability of 3-month good outcome (RR_unadjusted_ 0.970, 95% CI 0.950-0.989; RR_adjusted_ 0.975, 95% CI 0.956-0.995), and higher in-hospital mortality (RR_unadjusted_ 3.233, 95% CI 2.246-4.653; RR_adjusted_ 3.103, 95% CI 2.156-4.465), 1-month mortality (RR_unadjusted_ 1.070, 95% CI 1.048-1.093; RR_adjusted_ 1.064, 95% CI 1.042-1.087), and 3-month mortality (RR_unadjusted_ 1.075, 95% CI 1.051-1.099; RR_adjusted_ 1.069, 95% CI 1.045-1.093).

**Table 2:** Outcomes in patients admitted to hospitals during (December 1, 2019-November 30, 2020) and before (December 1, 2018-Novemver 30, 2019) the COVID-19 pandemic.

Characteristics	Time period		Unadjusted analysis		Adjusted analysis^a^	
	During the COVID-19 pandemic (n=1510), n (%)	Before the COVID-19 pandemic (n=1750), n (%)	Risk ratio (95% CI)	*P* value	Risk ratio (95% CI)	*P* value
Pulmonary infection	232 (15.4)	207 (11.8)	1.299 (1.091-1.546)	.003	1.267 (1.065-1.509)	.008
1-month mRS^b^ 0-3	797 (52.8)	977 (55.8)	0.980 (0.959-1.002)	.08	0.985 (0.963-1.007)	.17
3-month mRS 0-3	923 (61.1)	1158 (66.2)	0.970 (0.950-0.989)	.003	0.975 (0.956-0.995)	.01
In-hospital mortality	106 (7)	38 (2.2)	3.233 (2.246-4.653)	<.001	3.103 (2.156-4.465)	<.001
1-month mortality	282 (18.7)	190 (10.9)	1.070 (1.048-1.093)	<.001	1.064 (1.042-1.087)	<.001
3-month mortality	348 (23)	253 (14.5)	1.075 (1.051-1.099)	<.001	1.069 (1.045-1.093)	<.001

^a^Adjusted for age and gender.

^b^mRS: modified ranking scale.

## Discussion

Since December 2019, the outbreak of COVID-19 infections has had a major impact on the occurrence, development, and treatment of cerebrovascular diseases [7,8]. Currently, despite the international open policy, the epidemic still occasionally erupts in small areas. In this study, in order to investigate how the COVID-19 pandemic affected the manifestations and outcomes of patients with ICH, we retrospectively analyzed the data of patients with ICH in 1-year periods before (December 1, 2018-November 30, 2019) and during (December 1, 2019-November 30, 2020) the COVID-19 pandemic from the CHEERY study, a prospective multicenter cohort study. Our study found that during the COVID-19 pandemic, patients with ICH were more likely to be older with a history of ischemic stroke, higher blood pressure and fasting blood glucose, larger hematoma volume, shorter time from onset to admission, more common deep ICH and IVH, more ICU treatment, and preferred to go to hospital directly rather than call an ambulance. In addition, during the COVID-19 pandemic, patients with ICH had a higher risk of pulmonary infection, lower probability of 3-month good outcomes, and higher in-hospital, 1-month, and 3-month mortality.

ICH is among the diseases with the highest mortality; in low-income countries, its 30-day mortality may be as high as 40% [25]. Early identification and timely treatment are crucial to reducing patient mortality [26]. Research has found that more than one-third of patients have hematoma enlargement within 24 hours of onset, and approximately 38%-76% experience hematoma enlargement within 3 hours of onset. Every 10% increase in hematoma volume increases the risk of mortality by 5%, and every 1 mL increase in hematoma volume increases the risk of poor prognosis by 7% [27,28]. On the other hand, brain edema that occurs within hours of a patient’s onset and persists for days to weeks is also closely related to patient outcomes [29,30], with an average increase of 75% in perihematomal edema within the first 24 hours after ICH, which is also closely related to patient mortality [31,32]. In addition, persistent high systolic blood pressure in the early stages of ICH can lead to an increased risk of hematoma expansion, neurological deterioration, and even brain hernia formation, leading to patient death [33]. Therefore, early blood pressure reduction and intensive care for patients with ICH are crucial to reducing the mortality rate [34,35]. Unfortunately, such actions may have been difficult to implement during the pandemic.

During the pandemic, many studies reported a significant increase in the number of deaths from diseases other than COVID-19 [36,37]. Researchers found that in the early stages of the pandemic (March-July 2020), there was a marked increase in the number of patients dying from cerebrovascular disease [38]; this may have resulted from public health interventions [39] and limited medical care [40,41], patients refusing to enter the hospital for fear of infection [42,43], a decline in hospitals’ emergency care capacity [44,45], and insufficient poststroke intensive care [46] and rehabilitation care [10,47].

The global burden of ICH is related to the inadequate management of chronic hypertension and other modifiable risk factors [48]. In China, nearly half of adults aged 35-75 years have hypertension, but only 30.1% of patients with hypertension are being treated, and only about 7.2% are under control [49]. During the COVID-19 pandemic, the situation was worse, and the higher blood pressure in patients with ICH could be attributed to social pressure, anxiety, the depressed economy, the lack of public health resources, inadequate control of risk factors, and unwillingness to seek medical treatment during this special period [40-47]. Furthermore, the use of ACEI/ARBs might have theoretically increased the risk of COVID-19 infection, though recent reports did not find any correlation between the discontinuation of ACEI/ARB in patients with COVID-19 and the severity of COVID-19 [19,20]. For the fear of infection of COVID-19, patients may have discontinued the use of ACEI/ARB. A previous study in China also showed a significant increase in excess deaths due to cardiovascular disease and diabetes in the early lockdown period [43], presumably due to poor hypertension control during lockdowns in populations due to COVID-19 infections conflicting with antihypertensive drugs, such as ACEI.

Our previous studies have found that age, deep ICH, fasting blood glucose, and hematoma volume were predicters of in-hospital neurological deterioration and 3-month poor outcomes in the SIGNALS and ADVISING scores [50,51]. In this study, we found these factors were also associated with poor outcomes in patients with ICH during the COVID-19 pandemic. Older people had more risk factors, such as hypertension, diabetes mellitus, ischemic stroke, ischemic heart disease, and other diseases, which could induce and aggravate ICH. COVID-19 may have restricted the control of risk factors of ICH, resulting in more older patients with ICH.

Our study found that more patients with ICH went to hospitals directly without calling an ambulance. We speculate that this phenomenon was related to limited public medical resources and anxiety.

Most important of all, our study found that patients with ICH demonstrated higher NIHSS scores on admission and larger hemorrhage volumes. They also needed more ICU intervention and had poorer outcomes and mortality at 3 months. This finding indicated that ICH was more severe during the COVID-19 pandemic; this could be related to the patients’ higher blood pressure on admission, which may lead to a larger hematoma and poor prognosis. Our results were consistent with the report that older age and larger ICH volume are poor predictors of mortality [52].

One main limitation of our study was that we only included hospitalized patients. Those who were treated in an outpatient setting and died before reaching the hospital were not included. During the COVID-19 pandemic period corresponding to this study (December 1, 2019-November 30, 2020), more than 86,000 Chinese people were infected with COVID-19, as reported to the WHO. Vaccines, treatments, and minimally intrusive suppression measures are being developed for long-term prevention and control of COVID-19 in China [53]. As per government policy, infected patients were transferred to designated hospitals, which were not included in our study centers. Therefore, this study did not contain patients with COVID-19.

A major strength of this study is the use of the CHEERY study and its consecutive enrollment of patients within a defined study time, including the year before and after the COVID-19 outbreak. This excludes relevant selection bias and ensures that results from this cohort are fairly representative.

Our study indicated that the cloud of COVID-19 has adversely impacted the presentation and outcomes of ICH. Medical workers should pay more attention to patients with ICH, while the public should pay more attention to hypertension control and ICH prevention.

## Appendix

Multimedia Appendix 1 Data set.

## Abbreviations

ACEI: angiotensin-converting enzyme inhibitor
ARB: angiotensin receptor blockers
CHEERY: Chinese cerebral hemorrhage: mechanism and intervention
ICH: intracerebral hemorrhage
IVH: intraventricular hemorrhage
RR: risk ratio
SMASH-U: structural vascular lesions, medication, amyloid angiopathy, systemic disease, hypertension, and undetermined
STROBE: Strengthening the Reporting of Observational Studies in Epidemiology
WHO: World Health Organization

## Data Availability

Some of the data of this study are provided in Multimedia Appendix 1. Additional data are available upon reasonable request after approval by BH.

## References

[1] Huang C, Wang Y, Li X, Ren L, Zhao J, Hu Y, Zhang L, Fan G, Xu J, Gu X, Cheng Z, Yu T, Xia J, Wei Y, Wu W, Xie X, Yin W, Li H, Liu M, Xiao Y, Gao H, Guo L, Xie J, Wang G, Jiang R, Gao Z, Jin Q, Wang J, Cao B (2020). Clinical features of patients infected with 2019 novel coronavirus in Wuhan, China. The Lancet.

[2] Lau J, Yu Y, Xin M, She R, Luo S, Li L, Wang S, Ma L, Tao F, Zhang J, Zhao J, Hu D, Li L, Zhang G, Gu J, Lin D, Wang H, Cai Y, Wang Z, You H, Hu G (2021). Adoption of preventive measures during the very early phase of the COVID-19 outbreak in China: national cross-sectional survey study. JMIR Public Health Surveill.

[3] China. World Health Organization.

[4] Ke Y, Cui J, Wong Y (2021). Ecological study on differences in COVID-19 fatality among Wuhan, rest of Hubei, and rest of China. J Epidemiol Glob Health.

[5] Tang J, Abbasi K (2021). What can the world learn from China's response to covid-19?. BMJ.

[6] Pan A, Liu L, Wang C, Guo H, Hao X, Wang Q, Huang J, He N, Yu H, Lin X, Wei S, Wu T (2020). Association of public health interventions with the epidemiology of the COVID-19 outbreak in Wuhan, China. JAMA.

[7] Haldane V, De Foo C, Abdalla SM, Jung A, Tan M, Wu S, Chua A, Verma M, Shrestha P, Singh S, Perez T, Tan SM, Bartos M, Mabuchi S, Bonk M, McNab C, Werner GK, Panjabi R, Nordström A, Legido-Quigley H (2021). Health systems resilience in managing the COVID-19 pandemic: lessons from 28 countries. Nat Med.

[8] Tu W, Xu Y, Chen H, Li J, Du J (2023). Impact of the COVID-19 pandemic lockdown on hospitalizations for cerebrovascular disease and related in-hospital mortality in China: a nationwide observational study. Arch Gerontol Geriatr.

[9] Kansagra AP, Goyal MS, Hamilton S, Albers GW (2020). Collateral effect of Covid-19 on stroke evaluation in the United States. N Engl J Med.

[10] Rinkel LA, Prick JCM, Slot RER, Sombroek NMA, Burggraaff J, Groot AE, Emmer BJ, Roos YBWEM, Brouwer MC, van den Berg-Vos RM, Majoie CBLM, Beenen LFM, van de Beek D, Visser MC, van Schaik SM, Coutinho JM (2021). Impact of the COVID-19 outbreak on acute stroke care. J Neurol.

[11] Mariet A, Giroud M, Benzenine E, Cottenet J, Roussot A, Aho-Glélé LS, Tubert-Bitter P, Béjot Y, Quantin C (2021). Hospitalizations for stroke in France during the COVID-19 pandemic before, during, and after the national lockdown. Stroke.

[12] Zhao J, Li H, Kung D, Fisher M, Shen Y, Liu R (2020). Impact of the COVID-19 epidemic on stroke care and potential solutions. Stroke.

[13] Zhou Y, Hong C, Chang J, Xia Y, Jin H, Li Y, Mao L, Wang Y, Zhang L, Pan C, Hu J, Huang M, Wang D, Chen S, Hu B (2021). Intravenous thrombolysis for acute ischaemic stroke during COVID-19 pandemic in Wuhan, China: a multicentre, retrospective cohort study. J Neurol Neurosurg Psychiatry.

[14] Tanaka K, Matsumoto S, Nakazawa Y, Yamada T, Sonoda K, Nagano S, Hatano T, Yamasaki R, Nakahara I, Isobe N (2021). Delays in presentation time under the COVID-19 epidemic in patients with transient ischemic attack and mild stroke: a retrospective study of three hospitals in a Japanese prefecture. Front Neurol.

[15] GBD 2019 Stroke Collaborators (2021). Global, regional, and national burden of stroke and its risk factors, 1990-2019: a systematic analysis for the Global Burden of Disease Study 2019. Lancet Neurol.

[16] Hemphill JC, Greenberg SM, Anderson CS, Becker K, Bendok BR, Cushman M, Fung GL, Goldstein JN, Macdonald RL, Mitchell PH, Scott PA, Selim MH, Woo D (2015). Guidelines for the management of spontaneous intracerebral hemorrhage. Stroke.

[17] Qureshi AI, Palesch YY, Barsan WG, Hanley DF, Hsu CY, Martin RL, Moy CS, Silbergleit R, Steiner T, Suarez JI, Toyoda K, Wang Y, Yamamoto H, Yoon B (2016). Intensive blood-pressure lowering in patients with acute cerebral hemorrhage. N Engl J Med.

[18] Wan Y, Guo H, Shen J, Chen S, Li M, Xia Y, Zhang L, Sun Z, Chen X, Chang J, Wang D, He Q, Hu B (2022). Association between preonset anti-hypertensive treatment and intracerebral hemorrhage mortality: a cohort study from CHEERY. Front Neurol.

[19] Bauer A, Schreinlechner M, Sappler N, Dolejsi T, Tilg H, Aulinger BA, Weiss G, Bellmann-Weiler R, Adolf C, Wolf D, Pirklbauer M, Graziadei I, Gänzer H, von Bary C, May AE, Wöll E, von Scheidt W, Rassaf T, Duerschmied D, Brenner C, Kääb S, Metzler B, Joannidis M, Kain HU, Kaiser N, Schwinger R, Witzenbichler B, Alber H, Straube F, Hartmann N, Achenbach S, von Bergwelt-Baildon M, von Stülpnagel L, Schoenherr S, Forer L, Embacher-Aichhorn S, Mansmann U, Rizas KD, Massberg S (2021). Discontinuation versus continuation of renin-angiotensin-system inhibitors in COVID-19 (ACEI-COVID): a prospective, parallel group, randomised, controlled, open-label trial. Lancet Respir Med.

[20] Savarese G, Benson L, Sundström J, Lund LH (2021). Association between renin-angiotensin-aldosterone system inhibitor use and COVID-19 hospitalization and death: a 1.4 million patient nationwide registry analysis. Eur J Heart Fail.

[21] Zhou M, Guo W (2021). Social factors and worry associated with COVID-19: evidence from a large survey in China. Soc Sci Med.

[22] Han L, Zhan Y, Li W, Xu Y, Xu Y, Zhao J (2021). Associations between the perceived severity of the COVID-19 pandemic, cyberchondria, depression, anxiety, stress, and lockdown experience: cross-sectional survey study. JMIR Public Health Surveill.

[23] Mosconi MG, Paciaroni M, Agnelli G, Marzano M, Alberti A, Venti M, Acciarresi M, Ruffini F, Caso V (2021). SMASH-U classification: a tool for aetiology-oriented management of patients with acute haemorrhagic stroke. Intern Emerg Med.

[24] Chinese Clinical Trial Registry.

[25] van Asch CJ, Luitse MJ, Rinkel GJ, van der Tweel I, Algra A, Klijn CJ (2010). Incidence, case fatality, and functional outcome of intracerebral haemorrhage over time, according to age, sex, and ethnic origin: a systematic review and meta-analysis. The Lancet Neurology.

[26] Lord AS, Gilmore E, Choi HA, Mayer SA (2015). Time course and predictors of neurological deterioration after intracerebral hemorrhage. Stroke.

[27] Chang GY (2007). Hematoma growth is a determinant of mortality and poor outcome after intracerebral hemorrhage. Neurology.

[28] Wang W, Lu J, Liu L, Jia J, Zhao X (2021). Ultraearly hematoma growth in acute spontaneous intracerebral hemorrhage predicts early and long-term poor clinical outcomes: a prospective, observational cohort study. Front Neurol.

[29] Jiang C, Guo H, Zhang Z, Wang Y, Liu S, Lai J, Wang TJ, Li S, Zhang J, Zhu L, Fu P, Zhang J, Wang J (2022). Molecular, pathological, clinical, and therapeutic aspects of perihematomal edema in different stages of intracerebral hemorrhage. Oxid Med Cell Longev.

[30] Lee KH, Lioutas V, Marchina S, Selim M, iDEF Investigators (2022). The prognostic roles of perihematomal edema and ventricular size in patients with intracerebral hemorrhage. Neurocrit Care.

[31] Lv X, Li Z, Deng L, Yang WS, Li YL, Huang YJ, Shen YQ, Xie XF, Li XH, Wang ZJ, Zhang ZW, Lv FJ, Luo JB, Sun SJ, Xie P, Li Q (2021). Early perihematomal edema expansion: definition, significance, and association with outcomes after intracerebral hemorrhage. Oxid Med Cell Longev.

[32] Grunwald Z, Beslow LA, Urday S, Vashkevich A, Ayres A, Greenberg SM, Goldstein JN, Leasure A, Shi F, Kahle KT, Battey TWK, Simard JM, Rosand J, Kimberly WT, Sheth KN (2017). Perihematomal edema expansion rates and patient outcomes in deep and lobar intracerebral hemorrhage. Neurocrit Care.

[33] Mustanoja S, Putaala J, Koivunen R, Surakka I, Tatlisumak T (2018). Blood pressure levels in the acute phase after intracerebral hemorrhage are associated with mortality in young adults. Eur J Neurol.

[34] Li Q, Warren AD, Qureshi AI, Morotti A, Falcone GJ, Sheth KN, Shoamanesh A, Dowlatshahi D, Viswanathan A, Goldstein JN (2020). Ultra-early blood pressure reduction attenuates hematoma growth and improves outcome in intracerebral hemorrhage. Ann Neurol.

[35] Qureshi AI, Huang W, Lobanova I, Barsan WG, Hanley DF, Hsu CY, Lin C, Silbergleit R, Steiner T, Suarez JI, Toyoda K, Yamamoto H (2020). Outcomes of intensive systolic blood pressure reduction in patients with intracerebral hemorrhage and excessively high initial systolic blood pressure: post hoc analysis of a randomized clinical trial. JAMA Neurol.

[36] COVID-19 Excess Mortality Collaborators (2022). Estimating excess mortality due to the COVID-19 pandemic: a systematic analysis of COVID-19-related mortality, 2020-21. Lancet.

[37] Islam N, Shkolnikov VM, Acosta RJ, Klimkin I, Kawachi I, Irizarry RA, Alicandro G, Khunti K, Yates T, Jdanov DA, White M, Lewington S, Lacey B (2021). Excess deaths associated with covid-19 pandemic in 2020: age and sex disaggregated time series analysis in 29 high income countries. BMJ.

[38] Lee W, Woo Park S, Weinberger D, Olson D, Simonsen L, Grenfell BT, Viboud C (2023). Direct and indirect mortality impacts of the COVID-19 pandemic in the United States, March 1, 2020 to January 1, 2022. Elife.

[39] Teo K, Leung WC, Wong Y, Liu RK, Chan AH, Choi OM, Kwok W, Leung K, Tse M, Cheung RT, Tsang AC, Lau KK (2020). Delays in stroke onset to hospital arrival time during COVID-19. Stroke.

[40] Onteddu SR, Nalleballe K, Sharma R, Brown AT (2020). Underutilization of health care for strokes during the COVID-19 outbreak. Int J Stroke.

[41] Sheng S, Wang X, Gil Tommee C, Arulprakash N, Kamran M, Shah V, Jasti M, Yadala S, Brown A, Onteddu S, Nalleballe K (2021). Continued underutilization of stroke care during the COVID-19 pandemic. Brain Behav Immun Health.

[42] Lange S, Ritchey M, Goodman A, Dias T, Twentyman E, Fuld J, Schieve LA, Imperatore G, Benoit SR, Kite-Powell A, Stein Z, Peacock G, Dowling NF, Briss PA, Hacker K, Gundlapalli AV, Yang Q (2020). Potential indirect effects of the COVID-19 pandemic on use of emergency departments for acute life-threatening conditions - United States, January-May 2020. Am J Transplant.

[43] Liu J, Zhang L, Yan Y, Zhou Y, Yin P, Qi J, Wang L, Pan J, You J, Yang J, Zhao Z, Wang W, Liu Y, Lin L, Wu J, Li X, Chen Z, Zhou M (2021). Excess mortality in Wuhan city and other parts of China during the three months of the covid-19 outbreak: findings from nationwide mortality registries. BMJ.

[44] Laukkanen L, Lahtinen S, Liisanantti J, Kaakinen T, Ehrola A, Raatiniemi L (2021). Early impact of the COVID-19 pandemic and social restrictions on ambulance missions. Eur J Public Health.

[45] Fitzpatrick D, Duncan EAS, Moore M, Best C, Andreis F, Esposito M, Dobbie R, Corfield AR, Lowe DJ (2022). Epidemiology of emergency ambulance service calls related to COVID-19 in Scotland: a national record linkage study. Scand J Trauma Resusc Emerg Med.

[46] Wahlster S, Sharma M, Lewis A, Patel PV, Hartog CS, Jannotta G, Blissitt P, Kross EK, Kassebaum NJ, Greer DM, Curtis JR, Creutzfeldt J (2021). The coronavirus disease 2019 pandemic's effect on critical care resources and health-care providers: a global survey. Chest.

[47] Pop R, Quenardelle V, Hasiu A, Mihoc D, Sellal F, Dugay MH, Lebedinsky PA, Schluck E, LA Porta A, Courtois S, Gheoca R, Wolff V, Beaujeux R (2020). Impact of the COVID-19 outbreak on acute stroke pathways - insights from the Alsace region in France. Eur J Neurol.

[48] Schrag M, Kirshner H (2020). Management of intracerebral hemorrhage: JACC focus seminar. J Am Coll Cardiol.

[49] Lu J, Lu Y, Wang X, Li X, Linderman GC, Wu C, Cheng X, Mu L, Zhang H, Liu J, Su M, Zhao H, Spatz ES, Spertus JA, Masoudi FA, Krumholz HM, Jiang L (2017). Prevalence, awareness, treatment, and control of hypertension in China: data from 1·7 million adults in a population-based screening study (China PEACE Million Persons Project). The Lancet.

[50] He Q, Guo H, Bi R, Chen S, Shen J, Long C, Li M, Xia Y, Zhang L, Sun Z, Chen X, Wang Z, Gong D, Xu J, Zhu D, Wan Y, Hu B (2022). Prediction of neurological deterioration after intracerebral hemorrhage: the SIGNALS score. J Am Heart Assoc.

[51] Wan Y, Guo H, Chen S, Chang J, Wang D, Bi R, Li M, Shi K, Wang Z, Gong D, Xu J, He Q, Hu B (2023). ADVISING score: a reliable grading scale based on injury and response for intracerebral haemorrhage. Stroke Vasc Neurol.

[52] Poon MTC, Fonville AF, Al-Shahi Salman R (2014). Long-term prognosis after intracerebral haemorrhage: systematic review and meta-analysis. J Neurol Neurosurg Psychiatry.

[53] Li Z, Chen Q, Feng L, Rodewald L, Xia Y, Yu H, Zhang R, An Z, Yin W, Chen W, Qin Y, Peng Z, Zhang T, Ni D, Cui J, Wang Q, Yang X, Zhang M, Ren X, Wu D, Sun X, Li Y, Zhou L, Qi X, Song T, Gao GF, Feng Z, Li Z, Chen Q, Feng L, Rodewald L, Xia Y, Yu H, Zhang R, Yin W, Ni D, Qin Y, Zhang T, Cui J, Wang Q, Yang X, Zhang M, Chen W, Peng Z, Ren X, An Z, Wu D, Sun X, Li Y, Zhou L, Qi X, Gao GF, Feng Z, Song T, Luo H, Yin Z, Wang L, Ma C, Li S (2020). Active case finding with case management: the key to tackling the COVID-19 pandemic. The Lancet.

